# Effect of high-fat diet on the pharmacokinetics and safety of flumatinib in healthy Chinese subjects

**DOI:** 10.1007/s00280-020-04117-w

**Published:** 2020-08-05

**Authors:** Yun Kuang, Hui-ling Song, Guo-ping Yang, Qi Pei, Xiao-yan Yang, Ling Ye, Shuang Yang, Shu-ting Wu, Can Guo, Qing-nan He, Jie Huang

**Affiliations:** 1 Center of Clinical Pharmacology, The Third Xiangya Hospital, Central South University, Changsha, Hunan China; 2 Research Center for Drug Clinical Evaluation, Central South University, Changsha, Hunan China; 3 XiangYa School of Pharmaceutical Sciences, Central South University, Changsha, Hunan China; 4 Department of Pharmacy, The Third Xiangya Hospital, Central South University, Changsha, Hunan China; 5 Research Center for Drug Clinical Evaluation of Central, Central South University, Changsha, Hunan China; 6 Department of Pediatrics, The Third Xiangya Hospital, Central South University, Changsha, Hunan China

**Keywords:** Pharmacokinetics, Flumatinib, High-fat diet, Healthy subject

## Abstract

**Purpose:**

To evaluate the effect of a high-fat diet on the pharmacokinetics and safety of flumatinib mesylate tablets in healthy Chinese subjects.

**Methods:**

This study was a randomized, open-label, single-dose, two-period crossover trial in which subjects were randomly assigned to take 400 mg of flumatinib mesylate after a high-fat diet or a fasted state. After a 14-day washout period, the two groups were administered flumatinib mesylate under opposite conditions. Blood samples were collected at baseline 0 and 0.5, 1, 1.5, 2, 2.5, 3, 4, 5, 6, 8, 10, 12, 24, 48, 72, and 96 h, respectively. Plasma concentrations of flumatinib and its metabolites (M1 and M3) were analyzed using liquid chromatography-mass spectrometry. Pharmacokinetic parameters were calculated using the non-compartmental module of the Phoenix WinNonlin Version 7.0 software. BE module of WinNonLin was used for statistical analysis of AUC_0–t_, AUC_0–∞_ and *C*_max_ in plasma.

**Results:**

Twelve healthy subjects, half male and half female, were enrolled. One subject withdrew due to a treatment-emergent adverse event. Eleven subjects were administered drugs on fasting and 12 were administered drugs after a high-fat diet. On high-fat diet/fasting, the least square geometric mean (LSGM) ratios of flumatinib, M1, M3, and their 90% confidence interval (CI) were as follows: for flumatinib, C_max_, AUC_0–t_ and AUC_0–∞_ were 281.65% (225.80–351.31%), 167.43% (143.92–194.79%), and 166.87% (143.47–194.09%); for M1, *C*_max_, AUC_0–t_, and AUC_**0–∞**_ were 188.59% (145.29–244.79), 163.94% (149.11–180.24%), and 164.48% (150.36–179.94%); for M3, *C*_max_, AUC_0–t_, and AUC_0–∞_ were 63.47% (54.02–74.57%), 85.23% (74.72–97.22%), and 96.73% (86.63–108.02%).

**Conclusion:**

Among the subjects, oral administration of 400 mg of flumatinib was safe and well tolerated. High-fat diet significantly increases the exposure to flumatinib, therefore, fasting may be recommended.

**Clinical trial registration:**

The study was registered at chictr.org Identifier: ChiCTR-IIR-17013179.

## Background

Chronic myelogenous leukemia (CML) also called chronic granulocytic leukemia, is a slowly progressing blood and bone marrow disease that usually occurs during or after middle age, and rarely occurs in childhood [1]. A reciprocal chromosome translocation (9 and 22), called the Philadelphia chromosome, causes a constitutive activation of the BCR-ABL tyrosine kinase, leading to CML [2–5]. Current strategies for CML treatment involve the use of tyrosine kinase inhibitors, which can inhibit the BCR-ABL phosphorylation, thereby preventing the proliferation of cancer cells and activating subsequent apoptosis [2, 6–9]. Currently, resistance to the first-line drug imatinib has led to the development of other novel tyrosine kinase inhibitors [10].

Previous pharmacokinetic (PK) data of flumatinib showed that it was safe and well-tolerated, and Cmax and AUC_0–t_ were linearly related to doses in the range of 200–1000 mg [11]. Preclinical pharmacokinetic studies showed that, in rats and beagles, the maximum blood concentration could be reached in about 5 h after oral administration of the drug. Furthermore, flumatinib mesylate was widely distributed in tissues, with tissue drug concentration higher than that in plasma concentration. The parent drug flumatinib was present in plasma, urine, and feces. The primary metabolites in plasma were N-desmethyl flumatinib (M1), which was approximately 10% of that of the parent drug in plasma and has been shown to have similar pharmacological properties as the parent drug. The amide hydrolysis product (M3), which was inactive but approximately 30% of that of the parent drug in plasma [12]. Therefore, plasma concentrations of flumatinib, M1, and M3 were necessary to evaluate their circulating levels in humans.

Several studies have shown that gastrointestinal reactions, including abdominal pain, diarrhea, bloating, are the most common adverse reactions to tyrosine kinase inhibitors [13–16]. Therefore, tyrosine kinase inhibitors are often recommended to be administrated with food. However, food may affect gastric pH, emptying, and movement in the stomach, subsequently affecting drug absorption. In addition, since flumatinib is a lipophilic drug, and thus a high-fat diet may increase its solubility and (relative) bioavailability [17, 18]. Results from the Phase Ia clinical trials showed that the flumatinib absorption increased when administrated orally with food. However, as there are many influencing factors in patients with Phase Ia, it is important to evaluate the effect of food on the pharmacokinetics and safety of flumatinib mesylate in healthy subjects [19].

This study aimed to determine the effect of a high-fat, high-calorie diet on the pharmacokinetics of flumatinib and to evaluate the safety of oral administration of flumatinib 400 mg in healthy subjects.

### Methods

#### Subjects

Healthy Chinese subjects were screened for eligibility about 1 week before drug administration. Eligibility criteria included healthy Chinese adults, aged 18–45 years, body mass index (BMI) of 19–24 kg/m^2^, and a minimum weight of 50 kg. The subjects had no history of cardiovascular, endocrine, metabolic, neurological, gastrointestinal, hepatic, pulmonary, infectious, immunological, or psychiatric diseases. We excluded subjects with a history of alcohol abuse, cigarette or drug dependence, or under concomitant treatments defined as using any drug that inhibits/induces hepatic metabolizing enzymes, within 30 days, or having undergone a surgery in the last 4 weeks. In addition, female subjects who were pregnant, planning on conceiving, using oral contraception, or in the menstrual cycle were excluded from this study.

The study was approved by the Medical Ethics Committee of the Third Xiangya Hospital of Central South University (an independent data safety monitoring committee, certificated by the Association for the Accreditation of Human Research Protection Program). All participants provided written informed consent prior to any study-related procedure.

### Study design

This study was a single-center, randomized, open-label, two-period, crossover design to evaluate the effects of a high-fat diet on the pharmacokinetics of flumatinib in healthy subjects.

The subjects were randomized 24 h in advance in one of the two following groups. Group A: at least 10 h after fasting, oral flumatinib administration of 400 mg (2 tablets, day 1, first dose); the washout period was 14 days, half an hour after high-fat diet (timed from the start of diet) oral flumatinib administration of 400 mg (2 tablets, day 15,second dose). Subjects in group B followed the opposite administration sequence from those in group A. In groups A and B, the high-fat diet contained 800 ~ 1000Kcal (about 50% from fat) (meal composition is detailed Table 1).

**Table 1:** High-fat diet details*

Nutrients	Chinese oil sticks (100 g)	Egg (100 g)	Mixed oil (30 g)	Terunsu milk (250 ml)	Total (g)	Calories (KCal)	Percentage of calories(%)
Protein (g)	6.90	13.3	0	9.00	29.2	117	11.9
Carbohydrates (g)	50.1	2.80	0	12.5	65.4	261	26.5
Fat (g)	17.6	8.80	30.0	11.0	67.4	607	61.6
Total	74.6	24.9	30.0	32.5	162	985	100

*1 g protein was calculated by 4 kcal calories, 1 g carbohydrate by 4 kcal calories, and lg fat by 9 kcal calories. The Chinese oil sticks were calculated according to the finished products, and the eggs and mixed oil were calculated by raw food weight. Only the breakfast on the first day of each cycle was different. Other meals were totally the same for all the subjects in this trial

### Pharmacokinetic evaluations

Blood samples for pharmacokinetic (PK) evaluation were collected at 0 h before the initiation of dosing (pre-dose), and at 0.5, 1, 1.5, 2, 2.5, 3, 4, 5, 6, 8, 10, 12, 24, 48, 72, and 96 h after dosing. Blood samples were centrifuged at 2000*g* for 10 min at 4 ℃. Centrifugation was completed within 60 min after sample collection. The plasma was stored at − 70 °C for further analysis. Plasma concentrations of flumatinib and its main metabolites (M1 and M3) were determined using liquid chromatography-mass spectrometry (LC–MS/MS) [20].

### Safety evaluations

All subjects who participated in the study were included in the safety analysis. Safety was evaluated by vital signs, physical examination, ECG, laboratory examination, adverse events (AEs), and combined medication. All adverse events that occurred during the trial were classified into mild, moderate and severe levels based on NCI CTCAE v4.03.

### Statistical analysis

Phoenix WinNonlin Version 7.0 (Pharsight Corporation, Sunnyvale, CA, USA) software was used to calculate the pharmacokinetic parameters (*T*_max_, *C*_max_, AUC_0–t_, AUC_0–∞_, *t*_1/2_, V/F, CL/F) of flumatinib and its main metabolites using a non-compartmental method (NCA module). Linear Up Log Down trapezoidal method was used for AUC calculation. The BE module of WinNonLin was used to analyze AUC_0–t_, AUC_0–∞,_ and *C*_max_ of flumatinib in fed and fasting states after logarithmic conversion. The treatment group (fasting, high-fat diet), the treatment sequence (A, B), and treatment phase were fixed effects in the model, and the subjects nested in the sequence were random effects. The adjusted mean difference (fasting/high-fat diet) and its 90% confidence interval estimated by the model, were taken as the negative number to obtain the corresponding PK parameter geometric mean ratio (postprandial/fasting), to estimate its 90% confidence interval, and to evaluate the effect of a high-fat diet on the pharmacokinetics of flumatinib.

### Results

#### Subjects

**Fig. 1:**
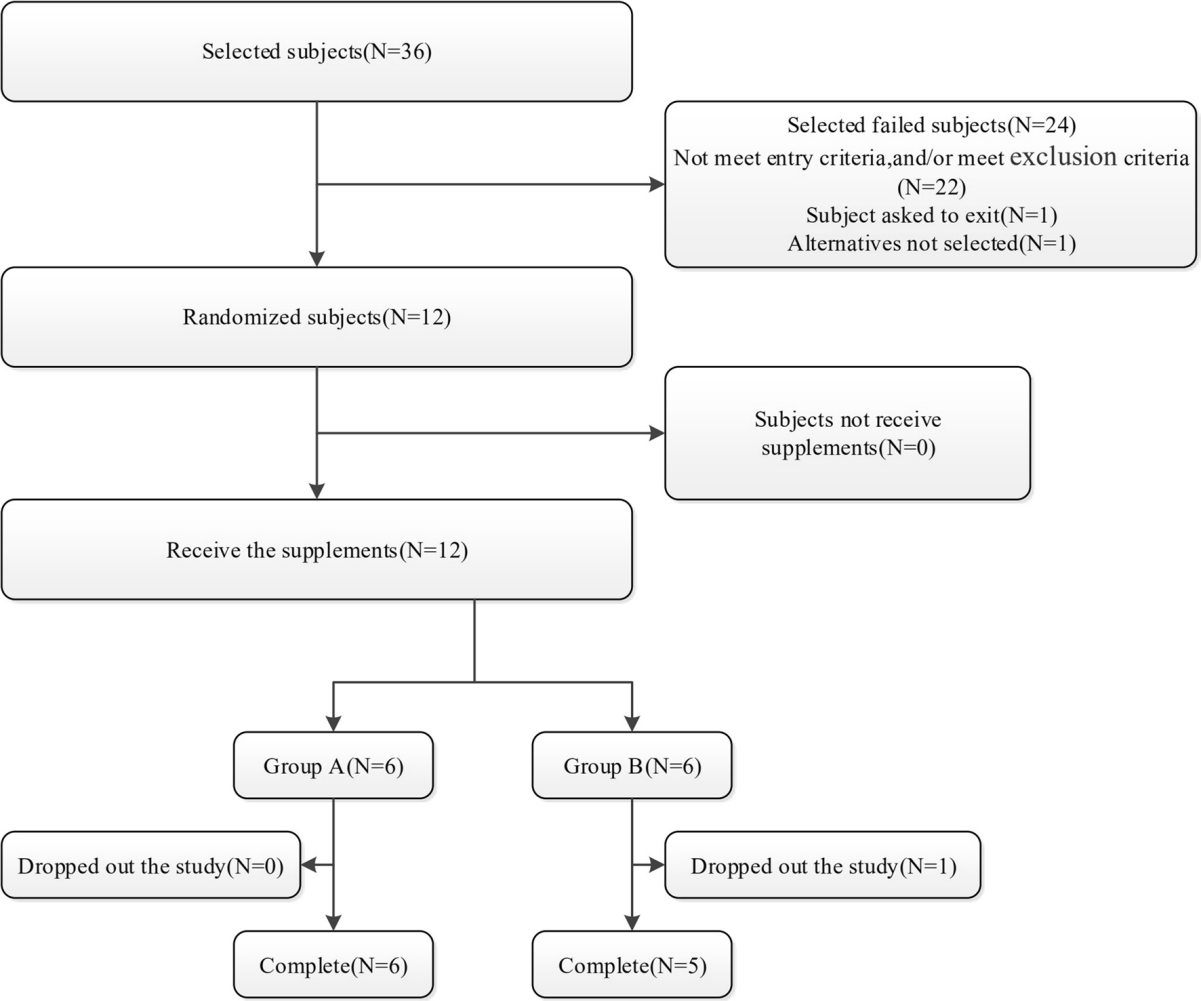
Consort diagram detailing the total number of subjects recruited, withdrawn, and analyzed

This study was conducted between May 8, 2017 and June 10, 2017. Thirty-six eligible patients were invited to participate in the study; a total of 12 patients agreed to be enrolled and were randomly assigned to group A or group B (*n* = 6 each). One subject in group B withdrew due to adverse events before the second period (Fig. 1). Half of the subjects were male and half were female. Age, height, weight, and BMI of the subjects were 23.5 ± 4.72 years, 165.0 ± 7.20 cm, 59.2 ± 5.10 kg, and 21.8 ± 1.92 kg/m^2^, respectively. The demographic and baseline characteristics of all subjects are presented in Table 2. Twelve subjects were involved in pharmacokinetics evaluations of high-fat diet and safety analysis, 11 subjects were included pharmacokinetics evaluations of fasting and the food effect on flumatinib pharmacokinetics.

**Table 2:** Demographic Information and Baseline Information of Subjects in Groups

Variables	Statistics	Group A	Group B	Total
Total number		6	6	12
Sex (%)				
Male		3 (50%)	3 (50%)	6 (50%)
Female		3 (50%)	3 (50%)	6 (50%)
Age, years	Mean (SD)	24.3 ± 6.12	22.7 ± 3.14	23.5 ± 4.72
Height, cm	Mean (SD)	165 ± 6.22	166 ± 8.64	165 ± 7.20
Weight, kg	Mean (SD)	60.0 ± 3.77	58.4 ± 6.43	59.2 ± 5.10
BMI, kg/m^2^	Mean (SD)	22.3 ± 2.05	21.3 ± 1.82	21.8 ± 1.92

Data are the mean ± SD, except sex (male/female), which is in %

*BMI* body mass index, *SD* standard deviation

### Pharmacokinetic evaluations

**Fig. 2:**
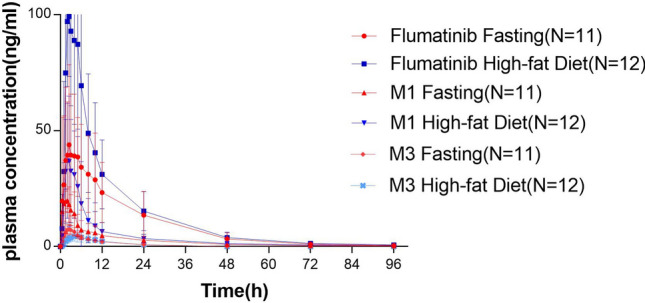
Mean plasma concentration of flumatinib, M1, and M3 in healthy Chinese subjects receiving a single oral 400 mg dose of flumatinib mesylate tablet

The mean plasma concentration versus time profiles of flumatinib, M1 and M3 in healthy Chinese subjects receiving a single oral dose of flumatinib mesylate tablet (400 mg) are shown in Fig. 2. The *C*_max_, AUC_0–t_, AUC_0–∞,_ and the secondary pharmacokinetic endpoints (*T*_max_, *T*_1/2_, V/F, CL/F and MRT) of flumatinib, M1 and M3 are shown in Table 3. Mean (± standard deviation) *C*_max_, AUC_0–t_, AUC_**0–∞**_ of flumatinib in plasma after a high-fat diet (132 ± 54.5 ng/ml; 1260 ± 582 ng h/ml; 1277 ± 586 ng h/ml, respectively) were higher than on fasting (50.7 ± 32.5 ng/ml; 832 ± 566 ng h/ml; 847 ± 572 ng·h/ml, respectively). M1 exposure was also increased in presence of a high-fat meal, C_max_, AUC_0–t_ and AUC_**0–∞**_ in plasma after high-fat diet (54.7 ± 25.5 ng h/ml; 368 ± 177 ng h/ml; 390 ± 191 ng h/ml, respectively) were higher than that after fasting (27.2 ± 7.14 ng/ml: 231 ± 101 ng/ml; 243 ± 108 ng h/ml, respectively). CL/F, V/F, and MRT in plasma after high-fat diet (661 ± 380 L/h; 11700 ± 5130 L; 19.2 ± 3.37 h, respectively) were lower than that after fasting (399 ± 229 L/h; 8470v± 4130 L, 17.2 ± 3.66 h, respectively).

**Table 3:** Pharmacokinetics Parameters of Fasting and High-fat Diet

Parameters	Flumatinib		M1		M3	
	Fasting (*N* = 11)	High-fat diet (*N* = 12)	Fasting (*N* = 11)	High-fat diet (*N* = 12)	Fasting (*N* = 11)	High-fat diet (*N* = 12)
*T*_max_ (h) median(range)	2.50 (1.50–5.00)	3.00 (1.50–5.02)	2.00 (0.50–4.00)	2.25 (1.50–5.02)	2.50 (1.50–4.00)	4.00 (2.00–10.00)
*C*_max_ (ng/mL)	50.7 ± 32.5	132 ± 54.5	27.2 ± 7.14	54.7 ± 25.5	7.95 ± 2.07	5.17 ± 2.21
AUC_0–t_ (ng.h/mL)	832 ± 566	1260 ± 582	231 ± 101	368 ± 176.6	68.6 ± 20.4	56.6 ± 21.9
AUC_0-∞_ (ng.h/mL)	847 ± 572	1277 ± 586	243 ± 108	390 ± 191	74.1 ± 19.5	66.1 ± 25.5
*t*_1/2_ (h)	13.3 ± 2.52	15.5 ± 4.67	22.8 ± 6.96	24.4 ± 5.80	8.34 ± 1.91	8.52 ± 2.40
CL/F (L/h)	661 ± 380	399 ± 229	/	/	/	/
V/F (L)	11,700 ± 5130	8470 ± 4130	/	/	/	/
MRT (h)	19.2 ± 3.37	17.2 ± 3.66	24.3 ± 4.37	23.5 ± 4.20	11.5 ± 2.52	14.0 ± 3.69

All data are given as the mean ± standard deviation

*AUC*_*0–t*_ area under the concentration–time curve from zero to the final measurable concentration, *AUC*_0–∞_ area under the concentration–time curve from time zero to infinity, *C*_*max*_ maximum concentration, *t*_*1/2*_ elimination half-life, *MRT* mean residence time

### Effect of high-fat diet on the pharmacokinetics

The least-square geometric mean (LSGM) ratio of flumatinib, M1, M3 and their 90% confidence interval (CI) after high-fat diet/fasting are shown in Table 4. The Cmax of flumatinib in plasma after a high-fat diet had nearly tripled over that in the fasted state, and the Cmax of M1 increased 1.9-fold. The AUC_0–t_ and AUC_0–∞_ of flumatinib and M1 increased 1.6-fold. Except for the 90%CI of M3 AUC_0–∞_ which was within 80.00–125.00%, the other parameters of flumatinib and metabolites M1 and M3 had 90% CI outside 80.00–125.00%. This indicated that a high-fat diet had a significant effect on pharmacokinetic of flumatinib, M1, and M3. And a high-fat diet can increase the peak concentration and systemic exposure of flumatinib and M1.

**Table 4:** Effect of high-fat diet on the pharmacokinetics of flumatinib, M1, and M3

Pharmacokinetics parameters	Flumatinib			M1			M3		
	Fasting (*N* = 11)	High-fat diet (*N* = 11)	LSGM ratio high-fat diet/fasting (%) (90% CI)	Fasting (*N* = 11)	High-fat diet (*N* = 11)	LSGM ratio high-fat diet/fasting (%) (90% CI)	Fasting (*N* = 11)	High-fat diet (*N* = 11)	LSGM ratio high-fat diet/fasting (%) (90% CI)
*C*_max_ (ng/mL)	43.7	123	282 (226, 351)	26.3	49.6	189 (145, 245)	7.69	4.88	63.5 (54.0, 74.6)
AUC_0–t_ (ng*h/mL)	696	1170	167 (144, 195)	212	347	164 (149, 180)	65.5	55.9	85.2 (74.7, 97.2)
AUC_0-∞_ (ng*h/mL)	710	1180	167 (143, 194)	223	367	164 (150, 180)	68.7	66.5	96.7 (86.6, 108)

*AUC*_*0–t*_ area under the concentration–time curve from zero to the final measurable concentration, *AUC*_0–∞_ area under the concentration–time curve from time zero to infinity, C_max_ maximum concentration

### Safety evaluations

Safety evaluations were performed in all subjects (*n* = 12). In the fasting group, there were nine cases of treatment-emergent adverse event (TEAE) (81.8%) and eight (72.7%) were related to the study drug. (Table 5). In the high-fat diet group, there were ten cases of TEAE (83.3%) and seven (58.3%) were related to the study drug. Except for one case of moderate acute gastroenteritis (withdrawal), the other TEAEs were mild. Acute gastroenteritis was completely relieved after treatments, including anti-infection, spasmolysis, antiemesis and acid suppression. Other mild adverse events disappeared without any treatment. The TEAE related to the drug included mild gastrointestinal symptoms (abdominal pain, diarrhea, and abdominal distension) and mild laboratory abnormalities (alanine aminotransferase, blood magnesium, and uric acid slightly increase).

**Table 5:** Summary of adverse events

Index	Fasting									High-fat diet								
	The first period (*N* = 6)			The second period (*N* = 5)			Total (*N* = 11)			The first period (*N* = 6)			The second period (*N* = 6)			Total (*N* = 12)		
	Time	Number	Incidence rate	Time	Number	Incidence rate	Time	Number	Incidence rate	Time	Number	Incidence rate	Time	Number	Incidence rate	Time	Number	Incidence rate
TEAE14	14	6	100.0	5	3	60.0	19	9	81.8	18	6	100.0	7	4	66.7	25	10	83.3
TEAE related to the study drug11	11	5	83.3	4	3	60.0	15	8	72.7	6	3	50.0	6	4	66.7	12	7	58.3
SAE related to the study drug0	0	0	0.0	0	0	0.0	0	0	0.0	0	0	0.0	0	0	0.0	0	0	0.0
TEAE related to death0	0	0	0.0	0	0	0.0	0	0	0.0	0	0	0.0	0	0	0.0	0	0	0.0
TEAE related to withdrawal0	0	0	0.0	0	0	0.0	0	0	0.0	1	1	16.7	0	0	0.0	1	1	8.3

*TEAE* treatment-emergent adverse event, *SAE* severity adverse even

### Discussion

**Fig. 3:**
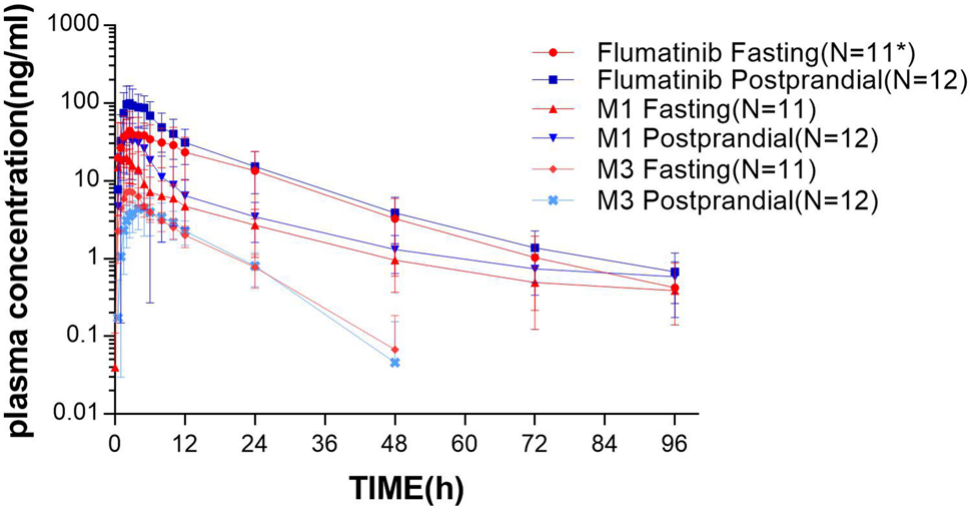
Semi-logarithmic plots of mean plasma concentration of flumatinib, M1 and M3 in chinese healthy subjects receiving a single oral 400 mg dose of flumatinib mesylate tablet

The results of the Phase Ia clinical trial showed that there were significant differences in AUC_0-t_ and C_max_ of flumatinib and M1 between fasting and high-fat diet (*p* < 0.05), implying the oral absorption of flumatinib could be promoted by postprandial administration. These are similar to the results of our study in healthy people. This is consistent with a report on a similar drug (ivosidenib), in which a 98% increase in Cmax was observed in healthy subjects who were given the drug after a high-fat diet, as compared to the fasting [21]. The increase in bioavailability of lapatinib after low-fat and high-fat diet were 167% and 325%, respectively [22]. The reasons for our results are as follows, First, a high-fat diet increases the secretion of bile acid (BA), especially secondary BA, such as deoxycholic acid and lithocholic acid, which slows gastric emptying and weakens intestinal peristalsis, resulting in prolonged retention time of flumatinib in the gastrointestinal tract, thereby increasing absorption [23]. Second, flumatinib is a lipophilic drug, thus, a high-fat diet can increase the solubility of flumatinib, thereby promoting drug absorption and increasing its (relative) bioavailability [24]. The *C*_max_ and AUC_0–t_ of M3 after a high-fat diet was decerased compared with that of fasting. This may be due to poor detection sensitivity as the M3 proportion is small. Alternatively, postprandial administration had less effect on the parent drug to M3 metabolic pathway. CL/F and V/F of flumatinib are huge because flumatinib has a low bioavailability. First, based on the BCS(Biopharmaceutics Classification System) Guidance of the year 2000, flumatinib is classified as BCS Class III. Second, a large amount of unmetabolized parent drug was detected in the feces after oral administration, which may be related to oral malabsorption caused by the low-permeability of flumatinib. Third, the bioavailability of flumatinib increased after high-fat meal, indicating that high-fat diet improved the low- permeability of flumatinib and improved the low-bioavailability of flumatinib, so the CL/F and V/F of postprandial administration decreased correspondingly (Fig. 3).

The incidence of TEAE was similar between the two conditions, while the incidence of TEAE related to the study drug after high-fat diet was better than that of fasting. However, it is uncertain whether the safety of the drug after a high-fat diet is better than that after fasting due to the small sample size. TEAEs including mild gastrointestinal symptoms and mild laboratory abnormalities are similar to most of the adverse reactions in oncology. For example, the FDA labels warnings of the potential for liver toxicity and rare cases of fatal liver failure [25].

The combination of a high-fat diet with flumatinib increased the bioavailability of flumatinib and M1. So taking it with food (which may increase its bioavailability) may have clinical implications, such as toxic reactions and increased accumulation. In this study, there was no significant increase in adverse reactions after a high-fat diet as compared with fasting. Combined with the clinical data of Phase II and III, the exposure and bioavailability of flumatinib after a high-fat diet was increased, so postprandial administration may have potential risks. The exposure and bioavailability of nilotinib, which is a BCR-ABL tyrosinase inhibitor, increased significantly after administration with food compared with fasting. In healthy subjects, *C*_max_ and AUC were reported to be 112% and 82% higher when taken with food than fasting [26]. Therefore, the marketing instructions for nilotinib indicate that it should be taken on an empty stomach. Therefore, in terms of the selection of the drug administration recommended in the later stage of clinical practice, flumatinib may only be taken on an empty stomach without food.

## Conclusion

Oral administration of flumatinib at a single dose of 400 mg was safe and well tolerated in healthy subjects on fasting or a high-fat diet. However, considering the significant increase (67%) in the exposure to flumatinib when taken with food, flumatinib may be recommended to be taken in the fasted state.
